# Fractality *à la carte*: a general particle aggregation model

**DOI:** 10.1038/srep19505

**Published:** 2016-01-19

**Authors:** J. R. Nicolás-Carlock, J. L. Carrillo-Estrada, V. Dossetti

**Affiliations:** 1 Instituto de Física, Benemérita Universidad Autónoma de Puebla, Puebla, 72570, Mexico; 2 CIDS-Instituto de Ciencias, Benemérita Universidad Autónoma de Puebla, Puebla, 72570, Mexico

## Abstract

In nature, fractal structures emerge in a wide variety of systems as a local optimization of entropic and energetic distributions. The fractality of these systems determines many of their physical, chemical and/or biological properties. Thus, to comprehend the mechanisms that originate and control the fractality is highly relevant in many areas of science and technology. In studying clusters grown by aggregation phenomena, simple models have contributed to unveil some of the basic elements that give origin to fractality, however, the specific contribution from each of these elements to fractality has remained hidden in the complex dynamics. Here, we propose a simple and versatile model of particle aggregation that is, on the one hand, able to reveal the specific entropic and energetic contributions to the clusters’ fractality and morphology, and, on the other, capable to generate an ample assortment of rich natural-looking aggregates with any prescribed fractal dimension.

From the formation of mineral veins, complex biopolymers to clusters of galaxies, aggregation phenomena are out-of-equilibrium processes of fractal pattern formation ubiquitous in nature^1^,^2^,^3^. Since the appearance of the diffusion-limited aggregation^4^ (DLA) and ballistic aggregation^5^ (BA) models, a plethora of studies have been developed trying to understand the ultimate aspects of the aggregation dynamics that give rise to *self-affine* or fractal clusters, the relationship of this *fractality* with their physical and chemical properties, and the most effective methods and techniques to controll fractal growth^6^,^7^,^8^.

Furthermore, these simple paradigmatic models, proposed as way to understand aggregation under *short-range* interactions, have contributed to reveal that the main sources of fractality in particle-cluster aggregation are related to the general entropic and energetic characteristics of the system. That is, when long-range interactions are negligible, the entropic information of the growing medium (such as its temperature and viscosity), encoded in the mean squared displacement of aggregating particles, is the main element of the dynamics that determines the fractality and morphology of the clusters. For example, random trajectories of the wandering particles in DLA generate branching clusters with fractal dimension *D* < *d*, where *d* is the dimension of the embedding space, whereas ballistic (straight line) trajectories in BA generate compact clusters with *D* = *d*^9^,^10^,^11^,^12^,^13^,^14^. On the other hand, when attractive or repulsive interactions can no longer be disregarded, aggregation dynamics can become quite complex. Nonetheless, experimental reults^15^,^16^,^17^,^18^,^19^ and computational models^20^,^21^,^22^,^23^,^24^,^25^,^26^,^27^,^28^ have shown that for short-range repulsive interactions, clusters tend to be compact with *D* ≈ *d*. Conversely, long-range attractive interactions generate highly ramified clusters with a non-trivial fractal behavior characterized by 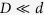, as the range of the interactions becomes larger.

In the last case, fractality is enhanced by the branching growth process that emerges from screening effects generated by the aggregated particles^29^,^30^, a fact that has led to consider that the main contribution to the fractality and morphology of the clusters is of an energetic character only, making the entropic one of no special significance but just as an intrinsic stochastic element of the dynamics^20^,^21^,^22^,^23^,^24^. However, while screening and anisotropic effects might play an important role when interactions are present^31^,^32^,^33^,^34^,^35^,^36^,^37^, in this Article we show that the entropic contribution cannot be trivially considered as this intrinsic stochastic element, but as an important aspect of the dynamics that contributes greatly to the fractality of the clusters, making it also a remarkable source of shape and texture in fractal pattern formation. To this end, trough the incorporation of an effective interaction or aggregation range^20^,^37^, *λ*, in the dynamics of the standard two-dimensional off-lattice particle-cluster DLA and BA models, we introduce a simple but non-trivial stochastic scheme that allows one to separate and characterize the subtle contributions of entropic and energetic character of the dynamics to the fractality and morphology of the clusters. This scheme also allows one to generate fractal clusters with rich morphological features, and most important, to harness absolute control over their fractal dimension.

The aggregation dynamics of our model is quite simple as illustrated in Fig. 1. In this work, we considered identical particles of a diameter equal to one, all distance quantities are measured in this particle-diameter units and, in order to characterize the fractality of the aggregates, we used the fractal dimension, *D* = *D*_*β*_, defined through the radius of gyration, *R*_*g*_ = *kN*^*β*^, where *k* is a constant, *N* is the number of particles in the cluster, and *β* = 1/*D*_*β*_. This quantity is estimated from the numerical derivative of *R*_*g*_(*N*) in logarithmic scale and averaged over a large ensemble (see Methods). Finally, we will refer to the known fractal dimension of the DLA or BA aggregates as *D*_0_ = 1.71 or 2, respectively.

## Results

### Aggregation under a constant interaction-range

For *λ* = 1, or *direct-contact* interaction, we obviously recover the usual DLA or BA models, as shown in Figs 2a and 3a, respectively (see also the Supplementary Animations S1 and S2 online). When *λ* > 1, interactions modify the local morphology of the aggregates, leading to a more stringy structure. However, two well defined features emerge due to the interplay of the long-range interactions and the way particles approach the cluster (in relation with their trajectories): a *multiscaling* branching growth and a crossover in fractality, from *D* → 1 (as *λ* → ∞) to *D* = *D*_0_ (when *N* → ∞), as shown in Figs 2b,c and 3b,c.

It can be appreciated that this growth presents three well defined stages. In the first one, the growth is limited by the interactions and is characterized by *D* → 1 as *λ* → ∞. This is due to the fact that the radial size of the cluster is small compared to *λ*. In consequence, the individual interaction regions of the aggregated particles are highly overlapped, forming an almost circular envelope or effective boundary of aggregation around the cluster (see Figs 2d and 3d). This makes the last (outermost) aggregated particles the most probable aggregation points in the cluster for the next incoming particle. Because of this, there is a tendency for the clusters to develop three main arms, clearly seen as *λ* → ∞ (see Figs 2e and 3e). This structural feature is reminiscent of a mean-field (MF) behavior^21^, characterized by *D* = 1, with three well defined branches. In the second stage, clusters exhibit a transition in growth dynamics. Here, the envelope starts to develop small deviations from its initial circular form, with typically three main elongations or growth instabilities associated with the main branches. When the distance between the tips of two adjacent branches becomes of the order of *λ*, a bifurcation process begins, generating multiscaling growth. Then, when the interactive envelope develops a branched structure itself, particles are able to penetrate into the inner regions of the aggregate and another transition in growth dynamics takes place, from *interaction-limited* to *trajectory-limited*. In the third stage, when the distance among the tips of the main branches becomes much larger than *λ*, growth is limited by the mean squared displacement of the wandering particles. In this case, the asymptotic value *D* = *D*_0_ as well as the main features in the global structure of the cluster are remarkably recovered as *N* → ∞, inheriting the main characteristics of the aggregation model used, either DLA or BA. That is, even though interactions leave a strong print in the local structure and fractality of the clusters, the stochastic nature of the particle trajectories will ultimately determine their global characteristics. Nonetheless, clusters cannot be trivially described by a single fractal dimension as it was previously thought^20^,^37^, since this multiscaling behavior is able to span many orders of magnitude in the space they occupy.

The previously described growth stages can be clearly observed in the behavior of *D* for *λ* = 100 and 1000, in Figs 2c and 3c (see also the Supplementary Animations S3 and S4 online). Even though the same crossover is present for smaller values of *λ*, the effects of the interactions in the first stage of growth are masked by the *small-cluster*-size regime. Furthermore, in both DLA and BA cases, the number of aggregated particles required to recover their typical fractal and morphological global behavior is directly proportional to the range of the interaction. On the other hand, as expected, the structural and fractal features will tend towards a MF behavior (despite the particle trajectories) when *λ* → ∞.

### Aggregation under a scaling interaction-range

The previous results have an important consequence. When *long-range* attractive interactions are introduced in the growth dynamics, the only way to obtain self-affine clusters, that is, clusters with a single fractal dimension, is to maintain a proper balance of energetic and entropic contributions in the growth process. In fact, taking into account that the spatial size of the clusters is proportional to the radius of gyration *R*_*g*_ ∝ *N*^1/*D*^, the desired balance can be achieved by scaling the interaction range with the number of particles in the cluster through the generalized *λ*(*N*) = *λ*_0_*N*^*ε*^. Here, *λ*_0_ is fixed to one, while *ε* is the scaling parameter that takes values in [0, 1]. As shown in Fig. 4a,b, given a fixed value of *ε*, this choice for *λ*(*N*) corrects for the multiscaling behavior of log *R*_*g*_, previously observed for a constant interaction and, remarkably, every aggregate grown under the scaling interaction proposed has a precise and uniquely defined fractal dimension *D* = *D*(*ε*). In fact, by computing *D*(*ε*) for different values of *ε*, one can define the ratios *f*_*s*_ = (*D*(*ε*) − 1)/(*D*_0_ − 1) and *f*_*E*_ = 1 − *f*_*s*_, that respectively quantify the specific entropic and energetic contributions to the fractal dimension of the clusters (see Fig. 4c,d). Here, one can clearly appreciate the transition in growth regimes from entropic, when *ε* → 0, to energetic, as *ε* → 1, and the non-trivial interplay between them to generate clusters with a specific fractal dimension; for the case of a constant and finite *λ*, the behavior of *f*_*s*_ and *f*_*E*_ is nontrivial due to the multiscaling characteristics of clusters, however, *f*_*s*_ → 1 and *f*_*E*_ → 0 as *N* → ∞. This important finding allows one to estimate the inverse of the relation, *ε*(*D*), in order to grow aggregates with any prescribed fractal dimension *D* in [1, *D*_0_], once the underlying DLA or BA model is selected as shown in Fig. 4e,f (see Methods). As far as we know, this control over the fractal dimension of the clusters and the range it spans, as well as over the morphology of the clusters, has not been obtained before under any other related scheme of tunable fractality^36^,^37^,^38^,^39^ or morphology^40^,^41^,^42^,^43^.

## Discussion

As previously stated, the entropic and energetic elements are two aspects of the complex aggregation dynamics which in nature are closely correlated. Nonetheless, this reductionist approach of encapsulating the information of all the finer details of the dynamics into an effective interaction or aggregation range has proven to be quite rewarding as one can appreciate in the diagram presented in Fig. 5, where we generically describe the whole family of fractal clusters that can be generated under this simple scheme of aggregation. Here, we shall recall that in two-dimensional systems (*d* = 2), by changing the fractal dimension of the particle trajectories, *d*_*w*_, from *d*_*w*_ = 2 (random) to 1 (ballistic), one can generate the whole set of clusters between *D* = 1.71 (DLA) to 2 (BA)^9^,^10^, this corresponds to the regime *λ* = 1 in Fig. 5. However, we are no longer restricted to purely entropic models of fractal growth (*λ* = 1) as, with the introduction of the energetic character provided by the interaction-range *λ*(*N*), we can now explore the full range of clusters with fractal dimensions in [1, *D*_0_]. Nonetheless, the purely entropic models (DLA or BA) have two important contributions to the clusters’ structure: first, they establish an upper limit to the fractal dimension (*D*_0_) and, second, they define a characteristic morphology for the clusters (that is of DLA or BA) as shown in Fig. 4f. Additionally, we are not necessarily bound to *d* = 2, since our model can be easily extended to higher dimensions^44^.

Furthermore, we have some interesting results regarding fractal universality. On one side, for a constant interaction range and only in the thermodynamic limit (*N* → ∞), we have aggregates that will remain within the same universality class (that is, same fractal dimension *D*_0_ and growth process^45^,^46^) corresponding to the underlying non-interactive aggregation model used to generate them, which can be BA, DLA or anything in-between. In this case, the entropic character of the dynamics is the main element that defines the universality class of clusters. However, when the condition *N* → ∞ is not fulfilled, clusters with the same number of particles will present different fractal characteristics, as the growth process is highly dependent on the range of *λ* (Figs 2b,c and 3b,c). On the other hand, when the interaction range is properly scaled with the size of the cluster, the scaling parameter *ε*(*D*), that keeps all the information about the energetic contribution of the dynamics, defines the universality class of the aggregates. In this case, even though the stochastic character of particle trajectories of the aggregation model used (BA, DLA or anything in-between) determines the upper range *D*_0_ of possible fractal dimensions in [1, *D*_0_] and the global morphological features of the clusters, the energetic character of the dynamics contained in *ε*(*D*) ultimately controls the growth process and therefore, defines the whole family of clusters belonging to the same universality class (Fig. 4a,b).

It is worth to mention that the model we propose remains quite simple in contrast to *Lagrangian* models (i.e., based on a molecular dynamics approach), where the cluster fractality comes from the stochastic and deterministic forces experienced by the particles^22^,^23^. Additionally, in contrast with the *screened-growth* or *sequential-algorithm* used to control the fractal growth in some other models^34^,^35^,^36^,^38^, we are now able to generate a remarkable range of multifractal and tunable fractal structures (once *ε*(*D*) is properly set), with no other parameters or pre-factors to determine.

Finally, because of its easy extension to higher dimensions and other spatial configurations, we anticipate that our model can be well exploited beyond aggregation phenomena, as it could provide important insights in the study of fractal growth phenomena, from fracture dynamics^47^, growth of bacterial colonies^48^,^49^, to networks^50^,^51^, as well as lead to novel applications in neuroscience^52^,^53^, complex materials^54^,^55^, and bio-inspired engineering^56^,^57^, among others.

## Methods

### Attractive interactions and aggregation dynamics

**Figure 1:**
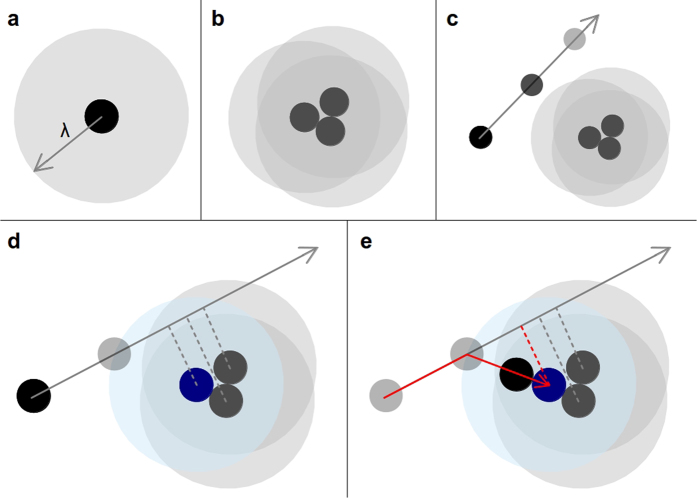
Implementation of the interaction. A step-by-step diagram is provided regarding the implementation of attractive interactions in our model. (**a**) Every particle in the cluster is provided with an effective radius of aggregation *λ*, starting with the seed particle. (**b**) Upon aggregation, the overlap between the independent interaction regions of the particles define an effective interaction boundary. (**c**) A particle far from this region does not interact with the cluster until (**d**) its trajectory is such that, for its next step, it intersects for the first time the interaction boundary of any aggregated particle. This is determined when its perpendicular distance to the particles in the cluster is less than *λ*. Then, the positions of the aggregated particles are projected to determine the closest one along the direction of motion. Finally, (**e**) the position of first crossing is computed and the position of the new particle in the cluster is determined.

The effective attractive interaction and aggregation dynamics in our model were implemented as explained in the discussion regarding Fig. 1. Additionally, *λ* can remain constant all along the dynamics or scale with *N*, depending on the desired application, i.e., to produce a multiscaling aggregate or an aggregate with a tuned fractal dimension. Further on, for aggregates based on BA, we follow the standard procedure in which particles are launched at random from the circumference of a circle of radius *L* = *r*_*max*_ + *δ*, with equal probability in position and direction of motion. Here, *r*_*max*_ is the distance of the farthest particle in the cluster with respect to the seed particle placed at the origin. In our simulations we used *δ* = 1000 particle diameters to avoid undesirable screening effects. In the case of aggregates based on DLA, particles were launched from a circle of radius *L* = *r*_*max*_ + *λ* + *δ*, with *δ* = 100. The mean free path for the motion of the particles is then set to one particle diameter, *λ*_0_ = 1. We also used a standard scheme that modifies (increments) the mean free path as the particles wander at a distances greater than *L* or in-between branches, and set a killing radius at *L*_*k*_ = 2*L*, in order to speed up the aggregation process.

### Evaluating the fractal dimension

**Figure 2:**
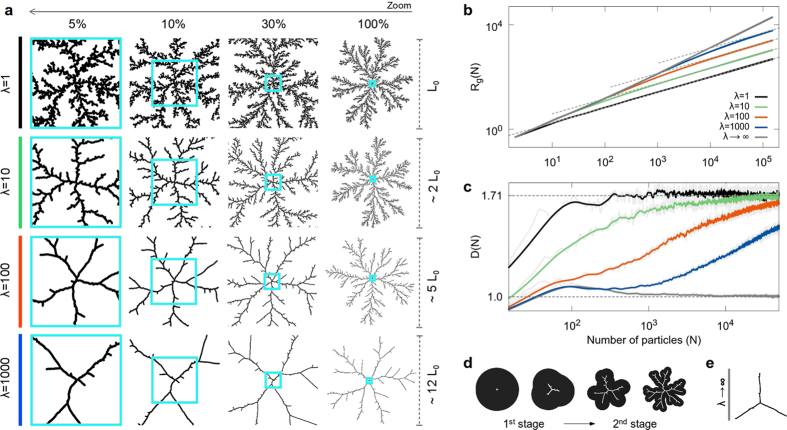
DLA-based multiscaling aggregates. (**a**) Aggregates containing *N* = 150 × 10^3^ particles each, for *λ* = 1, 10, 100 and 1000 units, visualized at 5%, 10%, 30% and 100% of their total size. The blue squares display the multiscaling evolution of the structure. (**b**) Radius of gyration, *R*_*g*_, and (**c**) fractal dimension, *D*, versus the number of aggregated particles, *N*, in log-log and lin-log plots, respectively. Notice that, when *λ* → ∞, the structure of the aggregates tends to MF (*D* = 1). Each curve was computed as an average over an ensemble of aggregates. (**d**) Evolution of the growing front for the first two stages of growth. (**e**) Typical structure of a MF aggregate.

**Figure 3:**
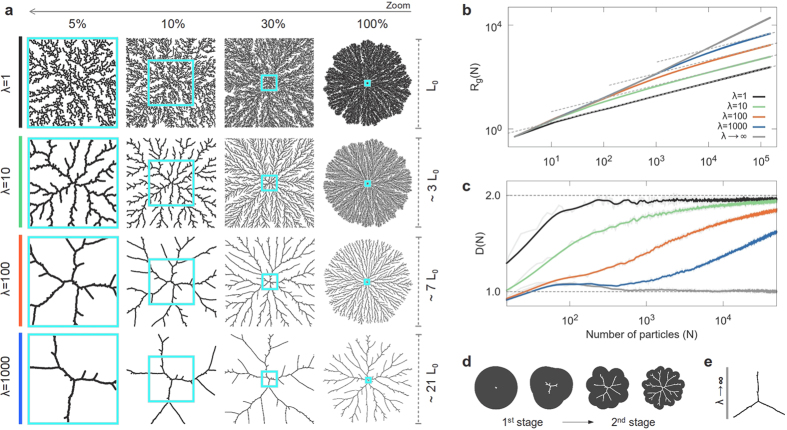
BA-based multiscaling aggregates. (**a**) Aggregates containing *N* = 300 × 10^3^ particles each, for *λ* = 1, 10, 100 and 1000 units, visualized at 5%, 10%, 30% and 100% of their total size. The blue squares display the multiscaling evolution of the structure. (**b**) Radius of gyration, *R*_*g*_, and (**c**) fractal dimension, *D*, versus the number of aggregated particles, *N*, in log-log and lin-log plots, respectively. Notice that, when *λ* → ∞, the structure of the aggregates tends to MF (*D* = 1). Each curve was computed as an average over an ensemble of aggregates. (**d**) Evolution of the growing front for the first two stages of growth. (**e**) Typical structure of a MF aggregate.

**Figure 4:**
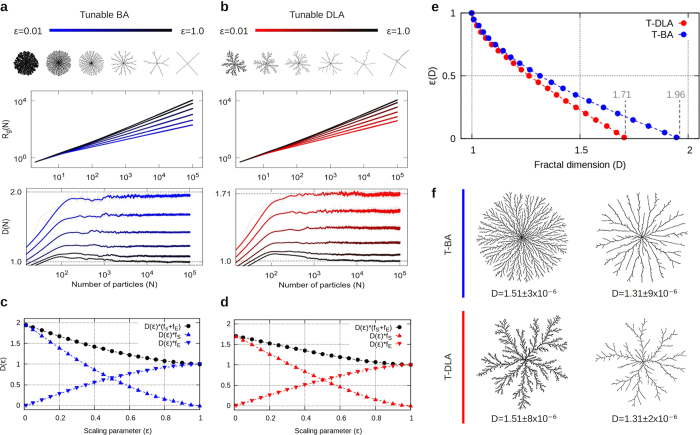
Tunable aggregates. (**a**,**b**) Log-log plots for *R*_*g*_(*N*) and lin-log for *D*(*N*), for aggregates grown with specific values of *ε* in [0.01, 1], for BA (blue) and DLA (red) up to *N* = 10^5^ particles. Notice how the multiscaling behavior gives way to a single well-defined fractal dimension *D* = *D*(*ε*). One can also appreciate the difference in the morphology of these monofractal-aggregates with respect to *ε* at the top. (**c**,**d**) Plots of the specific entropic [*D*(*ε*)*f*_*s*_] and energetic [*D*(*ε*)*f*_*E*_] contributions to the clusters’ fractal dimension *D*(*ε*) for BA and DLA as above. (**e**) Plots of *ε* vs. *D* for aggregates based on DLA and BA. These numerically obtained curves can be used to grow clusters with any prescribed *D*. (**f**) BA- and DLA-based clusters with the same fractal dimension, *D* = 1.51 and 1.31, grown with a very high precision in *D*.

**Figure 5:**
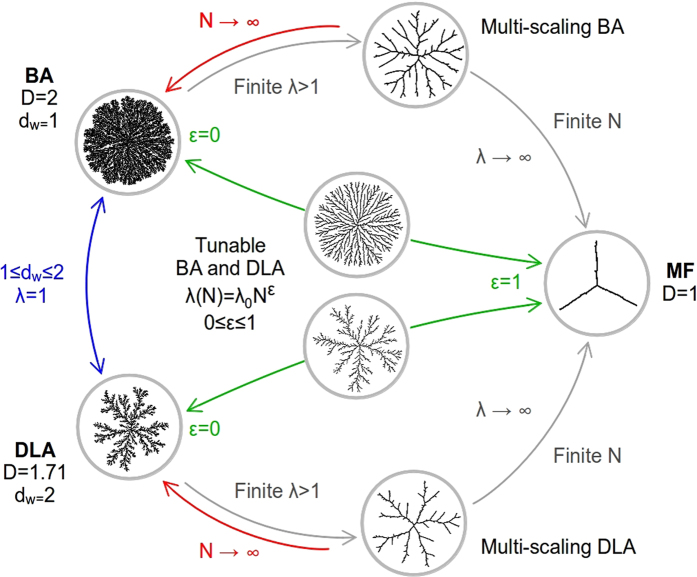
Fractality diagram. The diversity of aggregates that can be obtained with the introduction of *λ*(*N*) is astounding. (*i*) With *ε* = 0 and *λ*_0_ = 1, one has the well-known transition from BA to DLA by changing the mean squared displacement of the wandering particles, from ballistic to diffusive, respectively. (*ii*) With *ε* = 0 and a constant *λ*_0_ > 1 multiscaling aggregates are obtained, while MF behavior is obtained in the limit *λ* → ∞. Otherwise, for a finite *N*, one recovers usual BA or DLA behavior. Finally, (*iii*) with *ε* > 0 and *λ*_0_ = 1, aggregates with a tuned *D* from BA or DLA to MF, can be obtained by adequately scaling *λ* with *N*.

As it is usual in analyzing this kind of aggregates, the fractal dimension, *D*, is estimated from the radius of gyration, *R*_*g*_, by means of a linear-fit to *R*_*g*_ = *kN*^*β*^ in a log-log plot, where *k* is a constant, *N* is the number of aggregated particles and *β* = 1/*D*. In practice, it is assumed that *β* is constant as long as the number of particles in the cluster is large. Because the multiscaling models do not develop a constant fractal dimension at their early stages of growth, the simplest way to quantitative and qualitative characterize the behavior of *D* is through the derivative of *R*_*g*_ in the logarithmic scale. We did so by means of standard two and three point numerical differentiation methods: 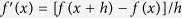, at the ends of the differentiation intervals and 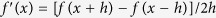, in between. Here *f*(*x*) = log *R*_*g*_(*N*) and *R*_*g*_(*N*) are computed as a discrete quantity therefore, *h* is set as the distance between the points, *x* = *x*_*j*_ and *x* + *h* = *x*_*j*+1_. In all cases, *R*_*g*_ is computed as an average over a large ensemble of aggregates. Specifically, the results for the multiscaling aggregates shown in Figs 2b and 3b, were obtained over 64 and 15 aggregates containing 1.5 × 10^5^ and 3 × 10^5^ particles for those based on DLA and BA, respectively. In this case, *R*_*g*_ was calculated every 10 particles. In Figs 2c and 3c, 192 and 128 aggregates containing 5 × 10^4^ and 10^5^ particles were used to obtain the averages of *D*(*N*), respectively, while *R*_*g*_ was calculated every 7 particles in order to capture the features of *D* at small scales in *N*. The results for tunable aggregates based on DLA and BA, shown in Fig. 4, were obtained over 128 and 48 aggregates, respectively, containing 10^5^ particles, and *R*_*g*_ was computed every 10 particles. We must point out that the fluctuations observed in *D*(*N*), depicted by the grey curves in Figs 2c, 3c and 4a,b, are due to the numerical and local aspect of the derivative’s estimation and the stochasticity of the model. These fluctuations decrease as *λ* increases, when the aggregates tend towards a better defined structure. Thus, with the purpose of improving the visualization of the observed tendencies, the curves in color were computed by means of a running-average over *N*.

### Tunable aggregates

Aggregates with a prescribed (tuned) fractal dimension, either based on BA or DLA, have a well defined *D* for each given value of *ε*. Therefore, we computed *D* for 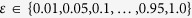 in order to obtain the functional dependence of *ε* on *D*. Then, for a desired D^*^, we estimated the corresponding value of *ε*(*D*) through a linear approximation using the two closest points in *D* to the desired D^*^. In this case, a linear approximation is a valid method since the difference among consecutive points in *ε*(*D*) is small and the curve is well behaved as can be appreciated in Fig. 4e., in these examples, the fractal dimension of the aggregates is estimated from a linear-fit to the plot of *R*_*g*_ in log-scale. All quantities are averaged over an ensemble of 128 clusters for each given value of *ε*.

## Additional Information

**How to cite this article**: Nicolás-Carlock, J. R. *et al.* Fractality *à la carte:* a general particle aggregation model. *Sci. Rep.* **6**, 19505; doi: 10.1038/srep19505 (2016).

## Supplementary Material

Supplementary Animation S1

Supplementary Animation S2

Supplementary Animation S3

Supplementary Animation S4
