# Supplementary figures and images for: Fractality à la carte: a general particle aggregation model
Source: Sci Rep. 2016 Jan 19;6:19505. doi: 10.1038/srep19505 (PMC4725996; doi:10.1038/srep19505)

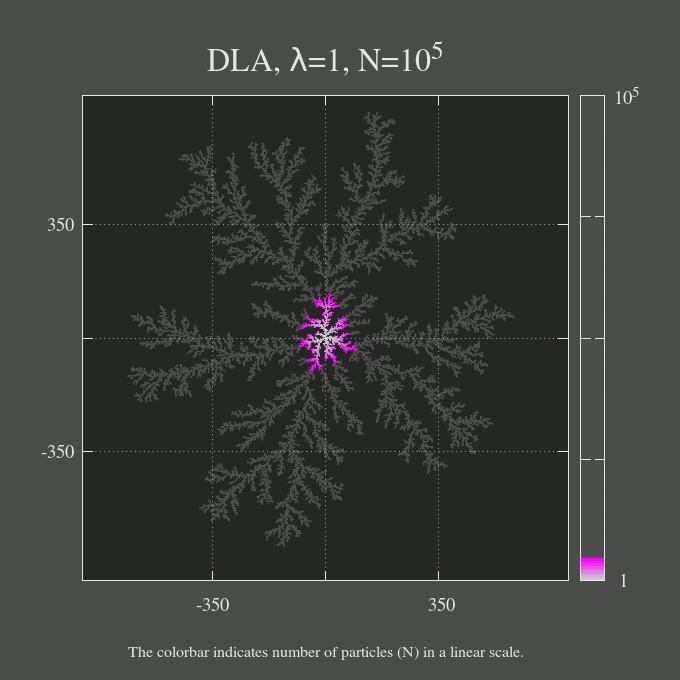

Supplement: Supplementary Animation S1 [file srep19505-s1.gif]

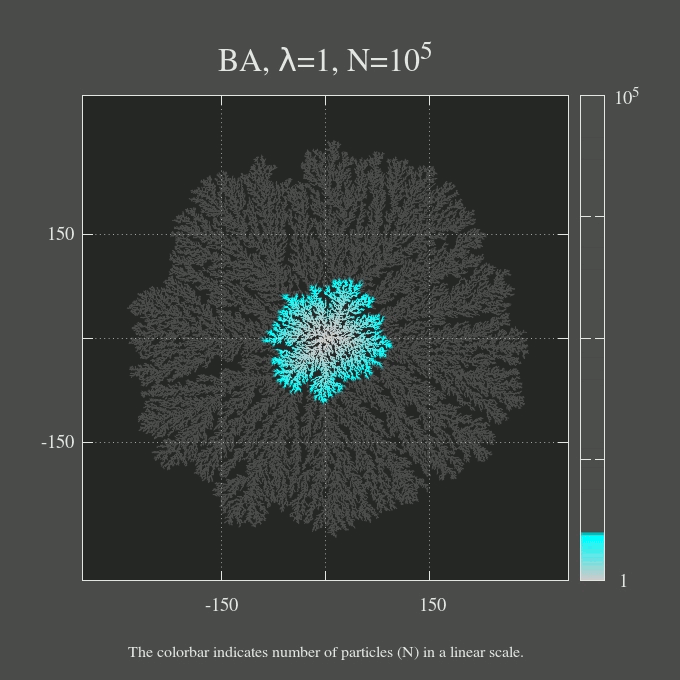

Supplement: Supplementary Animation S2 [file srep19505-s2.gif]

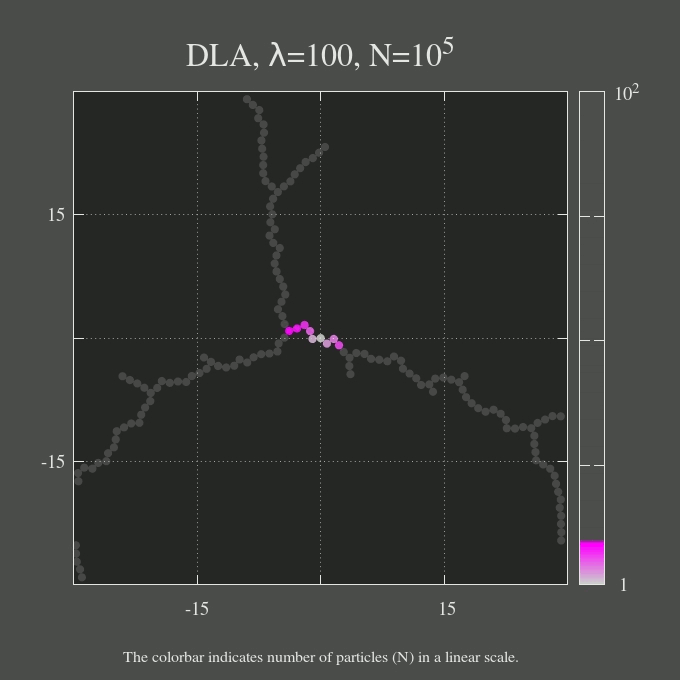

Supplement: Supplementary Animation S3 [file srep19505-s3.gif]

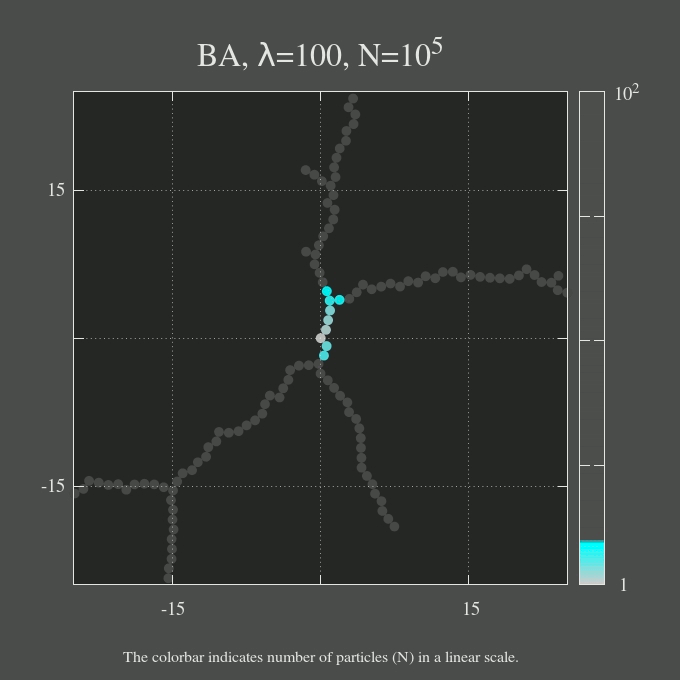

Supplement: Supplementary Animation S4 [file srep19505-s4.gif]
